# Genetic risk stratification reveals distinct first-order radiomic phenotypes in hepatocellular carcinoma

**DOI:** 10.3389/fonc.2026.1754407

**Published:** 2026-10-05

**Authors:** Mohamed Eltaher, Zaina Boriek, Rohan Amare, Awj Twam, Prasun Jalal, James Long, Eugene Koay, Khaled M. Elsayes, Rikita Hatia, Manal Hassan, David Fuentes

**Affiliations:** 1 The University of Texas MD Anderson Cancer Center, Houston, TX, United States; 2 Baylor College of Medicine, Houston, TX, United States

**Keywords:** genetics, hepatocellular carcinoma, personalized medicine, radiomics, tumor morphology

## Abstract

**Background:**

Hepatocellular carcinoma (HCC) exhibits significant morphological heterogeneity that has traditionally been attributed to etiological, environmental, and biological factors. Recent evidence suggests, however, that genetic background may influence tumor characteristics and behavior in HCC.

**Purpose:**

To investigate associations between established HCC genetic risk factors and first-order radiomic features.

**Methods:**

We evaluated a retrospective cohort of 210 patients with HCC who underwent contrast-enhanced MRI and genetic profiling for three established HCC risk variants (PNPLA3 rs738409-G, TM6SF2 rs58542926-T, and MAU2 rs58489806-T). We screened first-order radiomic features in the full cohort and then focused the primary analysis on 77 treatment-naive patients whose tumor morphology had not been modified by therapy. Patients were stratified into at-risk (≥1 risk allele, n=52) and wild-type (no risk alleles, n=25) groups. Eighteen first-order radiomic features were screened using the Mann-Whitney U test and AUC analysis. The leading feature, kurtosis, was then subjected to univariate and multivariate analysis for clinical and imaging features to identify any confounding effect. In addition, a 10-fold cross-validated cutpoint stability, sensitivity analysis, and Cox/Kaplan-Meier survival analysis were also conducted.

**Results:**

Among 18 features, kurtosis best distinguished at-risk from wild-type genotypes in treatment-naive patients (AUC = 0.67, p=0.014), with wild-type tumors showing more uniform, sharply peaked intensity patterns. Tumor volume correlated modestly with kurtosis (Spearman’s ρ=0.45, p<0.0001), but volume alone did not discriminate genotype (Mann-Whitney U, p=0.3994). The genotype-kurtosis association remained significant after adjusting for volume and shape (ANCOVA, p=0.029) and strengthened after adjusting for nine clinical/demographic covariates (p=0.003), indicating kurtosis reflects biological information beyond tumor size. The empirical cutpoint was stable across 10-fold cross-validation (mean=3.35, SD = 0.05), and results were unchanged in sensitivity analysis. Kurtosis was not associated with overall survival (Cox HR = 1.19, p=0.13). No genotype-related discrimination was observed in the pooled full cohort, consistent with treatment-related alteration of tumor texture.

**Conclusions:**

In a cohort of 210 genetically profiled patients with HCC, genotype-related radiomic features were not apparent when treated and untreated tumors were analyzed together. However, restricting the analysis to the treatment-naive subgroup (n=77) revealed tumor kurtosis as a reproducible, biologically plausible first-order imaging correlate of established HCC genetic risk status.

## Introduction

Hepatocellular carcinoma (HCC) represents the most common primary liver malignancy and ranks as the fourth leading cause of cancer-related mortality worldwide, with over 905,000 new cases diagnosed annually (1). Despite advances in surveillance, diagnosis, and treatment strategies, HCC continues to pose significant clinical challenges due to diverse clinical presentations, variable treatment response, and generally poor prognosis, with most cases diagnosed at advanced stages (2). The 5-year survival rate for early-stage HCC exceeds 70%, while advanced-stage disease carries a dismal prognosis of less than 20% survival over the same period, emphasizing the critical importance of early detection and targeted treatment approaches (3). HCC epidemiology has shifted over the past two decades. Viral hepatitis remains a global risk, but alcohol-related liver disease and MASLD are rising in Western countries due to lifestyle and demographic changes (2).

Recent large-scale genome-wide association studies (GWAS) have improved our understanding of HCC susceptibility by identifying specific genetic variants that significantly influence disease development. The three genes most significantly associated with HCC are MAU2 (MAU2 sister chromatid cohesion factor), TM6SF2 (transmembrane 6 superfamily member 2), and PNPLA3 (patatin-like phospholipase domain-containing 3) (4, 5). TM6SF2 and PNPLA3 are associated with MASLD and metabolic dysfunction-associated steatohepatitis (MASH), which are established risk factors for HCC, while the biological role of MAU2 in HCC development and its possible relationship to tumor histopathology are still under investigation.

Radiomics has emerged as a powerful tool for characterizing tissue morphology and predicting clinical outcomes (6, 7). These features encompass various aspects of tumor characteristics, including shape geometry, first-order statistical properties of intensity distributions, and higher-order textural patterns that reflect underlying biological processes.

The application of radiomics to HCC has shown promising results in various clinical contexts, including diagnosis, staging, treatment response prediction, and prognosis assessment (8). However, most studies have focused on clinical and treatment-related factors, with limited exploration of how genetic variation might influence imaging features.

Radiogenomics seeks to establish connections between imaging data and underlying genetic variants (9). In the context of HCC, this approach has the potential to improve understanding of how genetic background is expressed in tumor morphology.

Our primary hypothesis was that genetic risk variants influence HCC imaging phenotypes in measurable ways, and these relationships are modified after treatment.

## Methods

To test our hypothesis, we investigated associations between established HCC genetic risk factors and first-order radiomic features in a cohort that includes treatment-naive and treated patients, with a pre-specified analysis plan for testing confounding by tumor size, tumor shape, and clinical/demographic covariates, and for assessing the robustness and prognostic relevance of any association identified.

### Study design and patient population

This retrospective study included 210 patients with HCC from a previous GWAS cohort (4) who underwent both genetic profiling and contrast-enhanced magnetic resonance imaging (CE-MRI). The study was approved by the institutional review board, and informed consent was waived because of the retrospective design.

### Inclusion and exclusion criteria

Patients were included if they were at least 18 years old and had histologically or radiologically proven HCC, available genetic profiling data, and available high-quality CE-MRI scans suitable for radiomics analysis. Exclusion criteria comprised mixed hepatocellular-cholangiocarcinoma, concurrent other primary malignancies, MRI image artifacts, and missing genetic or clinical data.

### Imaging and clinical data collection

MRI studies were collected from electronic medical records; other patient data were retrieved from the GWAS study (4). Demographic variables included age at diagnosis, sex, mean body mass index (BMI), and race/ethnicity. Clinical risk factors were cirrhosis status as determined by histological, imaging, or clinical criteria; viral hepatitis status based on hepatitis B surface antigen and hepatitis C antibody positivity; alcohol consumption history, with significant use defined as greater than 14 drinks/week for men and 7 drinks/week for women; diabetes mellitus based on medical records or medication use; and family history of liver disease or HCC (**Table 1**).

**Table 1:** Patient characteristics stratified by genetic and treatment status; values are presented descriptively to summarize baseline subgroup composition.

Characteristic	At-risk untreated (n=52)	At-risk treated (n=82)	Wild-type untreated (n=25)	Wild-type treated (n=51)	Total (n=210)
Age, mean ± SD (range)	66.5 ± 9.7 (44-84)	65.7 ± 11.0 (23-87)	64.9 ± 10.9 (40-88)	65.3 ± 9.2 (41-85)	65.8 ± 10.2 (23-88)
Male sex, n (%)	42 (80.8%)	66 (80.5%)	15 (60.0%)	43 (84.3%)	170 (79.4%)
BMI, mean ± SD	27.9 ± 5.6	28.2 ± 4.7	27.5 ± 5.8	28.0 ± 5.1	28.0 ± 5.1
Cirrhosis, n (%)	35 (67.3%)	56 (68.3%)	11 (44.0%)	33 (64.7%)	138 (64.5%)
Alcohol history, n (%)	39 (75.0%)	60 (73.2%)	19 (76.0%)	45 (88.2%)	167 (78.0%)
Diabetes, n (%)	24 (46.2%)	33 (40.2%)	4 (16.0%)	11 (21.6%)	75 (35.0%)
Viral hepatitis, n (%)	15 (28.8%)	28 (34.1%)	12 (48.0%)	24 (47.1%)	80 (37.4%)
Tumor volume, mm³ ± SD	362,359 ± 524,065	337,677 ± 635,407	702,462 ± 909,871	280,229 ± 475,699	373,406 ± 619,203
Max diameter, mm ± SD	129.1 ± 82.8	114.9 ± 88.5	156.9 ± 76.9	111.8 ± 90.0	123.8 ± 91.5
Tumor count, mean ± SD	6.9 ± 17.1	5.1 ± 14.2	2.8 ± 4.9	6.3 ± 13.0	6.3 ± 18.5
Median follow-up, months (range)	18.4 (0-219)	23.4 (0-197)	13.8 (0-92)	24.4 (5-350)	21.0 (0-350)

Treatment status was carefully categorized to enable meaningful stratification. Treatment-naive patients (n=77) were defined as those who had not received any HCC-directed therapy prior to imaging. Treated patients (n=133) included those who had received any type of HCC treatment prior to the analyzed images, comprising liver-directed therapies such as transarterial chemo- or radioembolization or ablation (n=72); surgical resection (n=26); and systemic chemotherapy or immunotherapy (n=35).

### Genetic risk group classification

Based on the cumulative risk model established in the GWAS study (4), the cohort was stratified into two primary groups. The at-risk group (n=133, 63.3%) consisted of patients carrying at least one variant risk allele from any of the three high-risk genes (PNPLA3-G, TM6SF2-T, or MAU2-T). The wild-type group (n=77, 36.7%) included patients with no risk alleles from any of the three genes (homozygous for wild-type alleles). This binary classification was chosen to maximize statistical power while maintaining biological relevance, as previous studies have demonstrated that the presence of any risk variant allele substantially increases HCC susceptibility compared to the absence of all risk variant alleles. An overview of patient selection and workflow is shown in (**Figure 1**).

**Figure 1:**
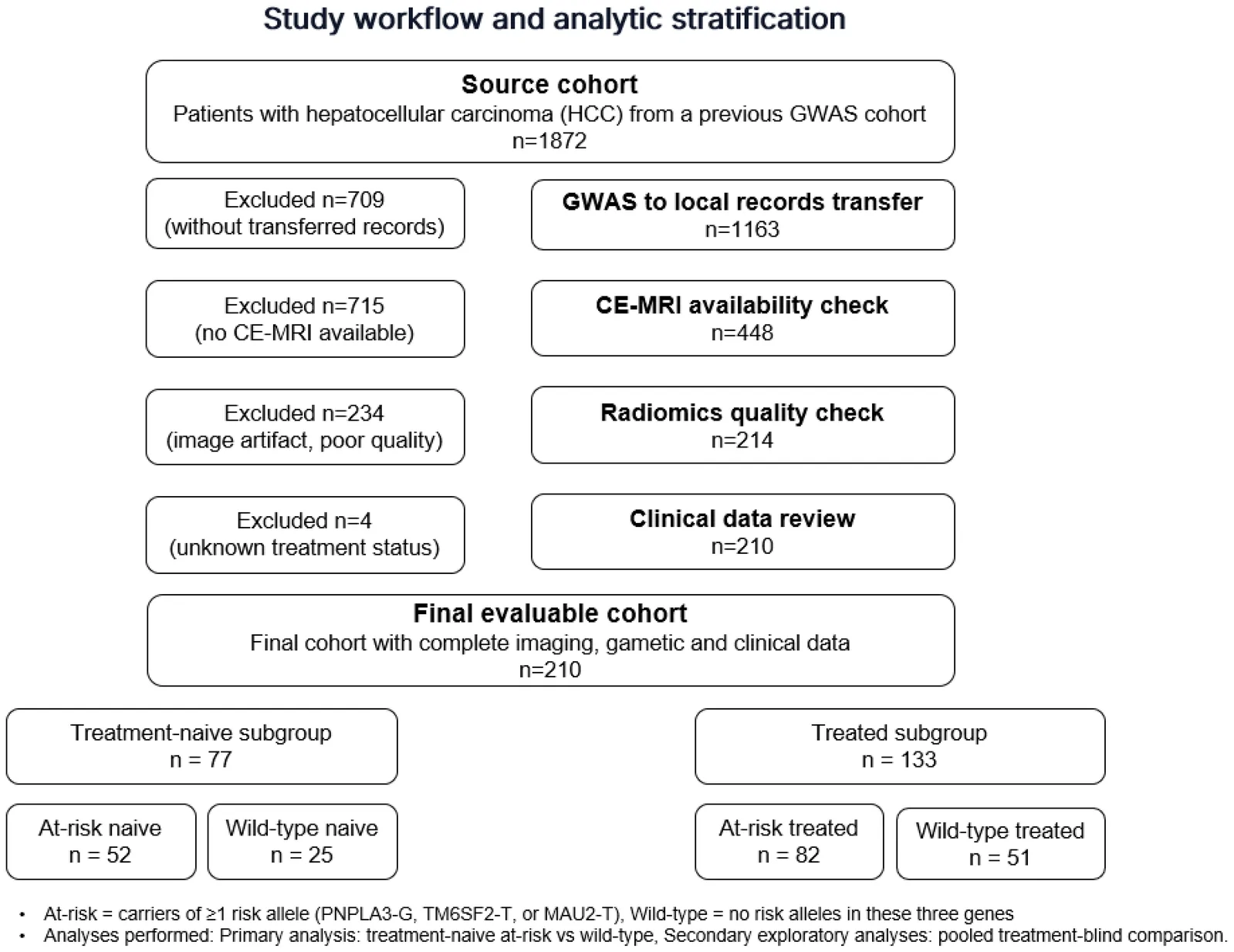
study workflow and analytical stratification.

### Imaging protocol

T1-weighted, contrast-enhanced, liver protocol MRI scans were collected for the cohort from the Picture Archiving and Communication System (PACS), independent of the patients’ treatment status. Magnetic resonance imaging (MRI) was performed using GE systems: a Signa Excite 1.5 T, Signa HDxt 1.5 T, Optima MR450w 1.5 T, and Discovery MR750 3.0 T (all GE Medical Systems, LLC [GE HealthCare], Waukesha, WI, USA), and MAGNETOM Aera 1.5 T system (Siemens Medical Solutions USA, Inc. [Siemens Healthineers], Malvern, PA, USA).

### Tumor segmentation and region of interest definition

In our study, tumor segmentation was performed by a resident radiologist guided by the radiology report for each scan through a systematic protocol beginning with initial identification of target lesions on arterial phase series showing characteristic HCC enhancement patterns and tumor remnants after treatment. Three-dimensional segmentation of volumetric regions of interest was performed manually using ITK-SNAP software (10). Quality control measures ensured that all segmentations were reviewed by a radiologist with over 20 years of experience in body imaging to maintain accuracy and consistency. Segmentations were standardized to exclude areas of central necrosis, vascular structures, and adjacent normal liver tissue. For patients with multiple lesions, the largest lesion was selected for analysis to maximize the reliability of radiomics feature extraction.

### Radiomics feature extraction

Radiomics analysis was performed using PyRadiomics (version 3.0.1) in compliance with Image Biomarker Standardization Initiative (IBSI) guidelines to ensure reproducibility and standardization. Feature extraction was performed on arterial-phase images due to their optimal tumor-to-liver contrast for characterizing HCC.

Prior to feature extraction, images underwent standardized preprocessing including resampling to isotropic voxel spacing (1×1×1 mm³), intensity normalization using z-score standardization, and discretization using the fixed bin width approach.

First-order statistical features (n=18) were extracted and comprised central tendency measures including mean, median, and mode; dispersion measures such as variance, standard deviation, and range; distribution shape measures including skewness and kurtosis; and intensity distribution at the 10th and 90th percentiles.

### Feature selection and dimensionality reduction strategy

To mitigate overfitting risk in the context of our sample size (n=77 treatment-naive patients and n= 133 treated patients were analyzed), we implemented a conservative feature selection strategy at multiple stages of the analysis pipeline.

During feature extraction, we limited our analysis to first-order statistical features (n=18) rather than extracting the complete PyRadiomics feature set, which includes hundreds of texture and filtered features. This hypothesis-driven approach was motivated by our biological interest in distribution characteristics that might reflect genetic influences on tumor development, specifically focusing on central tendency (mean, median, mode), dispersion (variance, standard deviation, range), and shape measures (skewness, kurtosis).

This multi-stage strategy effectively reduced dimensionality from the outset rather than extracting high-dimensional features and subsequently applying aggressive feature reduction, thereby minimizing the risk of spurious associations in our relatively modest sample size (11).

### Statistical analysis

#### Primary screening test

The Mann-Whitney U test (Wilcoxon rank-sum test) was used to assess statistically significant differences in each first-order feature between genetic risk groups, with Area Under the Curve (AUC) analysis providing complementary information on discriminative capacity (12). A p-value threshold of <0.05 was applied, with AUC interpreted relative to deviation from 0.5 (chance).

#### Tumor volume and shape confounding

Because first-order features can be mechanically influenced by the number of voxels contributing to the intensity histogram, we tested the Spearman correlation between tumor volume and kurtosis, and re-evaluated the genotype-kurtosis association after (1) residualizing log-kurtosis on log-tumor-volume, and (2) fitting a multivariable linear model (ANCOVA) with genotype group, log-tumor-volume, sphericity, and surface-to-volume ratio as simultaneous covariates. We additionally fit multivariable logistic regression models predicting elevated kurtosis (above the empirical threshold) from genotype groups, adjusted separately for log tumor volume and for maximum 3D diameter (13).

#### Clinical and demographic confounding

We fit a second ANCOVA model predicting log-kurtosis from genotype group plus nine clinical/demographic covariates simultaneously (sex, age, BMI, diabetes history, hepatitis C, hepatitis B core antibody status, smoking history, alcohol history, and cirrhosis) in patients with complete clinical data.

#### Cutpoint stability

Because the exploratory kurtosis threshold was derived and evaluated within the same cohort, we assessed its stability using 10-fold cross-validation, re-deriving the median-based cutpoint independently within each training fold and applying it to the corresponding held-out fold.

#### Survival analysis

We tested whether kurtosis and genotype groups were associated with overall survival using Cox proportional-hazards regression and Kaplan-Meier estimation.

All tests were two-sided with α=0.05. Statistical analyses were performed using Python 3.8+ with pandas (1.3+) and NumPy (1.20+), SciPy (1.7+) and statsmodels (0.12+) for statistical testing, scikit-learn (0.24+) for cross-validation, and matplotlib (3.3+) and seaborn (0.11+) for visualization.

## Results

### Primary finding

Among 18 first-order features screened in the pooled, treatment-blind cohort, kurtosis showed no meaningful discrimination (AUC = 0.45, p=0.25; **Table 2**), consistent with the expectation that mixing treated and untreated tumors dilutes any genotype-driven texture difference. Restricting to the treatment-naive subgroup, however, kurtosis emerged as the strongest single first-order discriminator between at-risk and wild-type patients (AUC = 0.67, p=0.014), with wild-type patients showing a more peaked voxel-intensity distribution than at-risk patients (**Figures 2**, **3**).

**Table 2:** AUC (p-value) for all 18 first-order features, at-risk vs. wild-type, tested treatment-blind (pooling all 210 patients regardless of treatment status) and separately within each treatment stratum.

Feature	Treatment-blind (n=210)	Treatment-naive (n=77)	Liver-directed (n=72)	Systemic (n=35)	Surgery (n=26)
Kurtosis	0.55 (0.254)	0.68 (0.014) *	0.54 (0.546)	0.57 (0.528)	0.64 (0.227)
InterquartileRange	0.51 (0.900)	0.63 (0.059)	0.51 (0.893)	0.62 (0.253)	0.71 (0.076)
90Percentile	0.53 (0.457)	0.63 (0.062)	0.67 (0.019) *	0.57 (0.484)	0.52 (0.898)
RobustMeanAbsoluteDeviation	0.51 (0.849)	0.63 (0.077)	0.51 (0.874)	0.62 (0.253)	0.71 (0.068)
MeanAbsoluteDeviation	0.51 (0.730)	0.62 (0.099)	0.52 (0.738)	0.62 (0.239)	0.70 (0.095)
Variance	0.52 (0.614)	0.61 (0.135)	0.53 (0.729)	0.63 (0.200)	0.68 (0.129)
Median	0.54 (0.313)	0.57 (0.341)	0.67 (0.021) *	0.54 (0.720)	0.52 (0.898)
RootMeanSquared	0.54 (0.281)	0.56 (0.364)	0.68 (0.014) *	0.55 (0.621)	0.54 (0.738)
Maximum	0.54 (0.315)	0.56 (0.411)	0.61 (0.110)	0.60 (0.331)	0.63 (0.269)
Mean	0.54 (0.327)	0.56 (0.424)	0.67 (0.017) *	0.53 (0.746)	0.55 (0.700)
Minimum	0.51 (0.782)	0.55 (0.468)	0.53 (0.718)	0.57 (0.499)	0.54 (0.737)
Entropy	0.52 (0.041) *	0.51 (0.594)	0.54 (0.061)	0.54 (0.214)	0.50 (1.000)
Uniformity	0.52 (0.041) *	0.51 (0.594)	0.54 (0.061)	0.54 (0.214)	0.50 (1.000)
10Percentile	0.54 (0.379)	0.54 (0.598)	0.66 (0.030) *	0.53 (0.798)	0.61 (0.368)
Skewness	0.53 (0.453)	0.53 (0.636)	0.54 (0.546)	0.64 (0.167)	0.58 (0.520)
Energy	0.53 (0.439)	0.52 (0.732)	0.60 (0.151)	0.53 (0.772)	0.51 (0.939)
TotalEnergy	0.52 (0.611)	0.51 (0.849)	0.60 (0.165)	0.53 (0.746)	0.51 (0.939)
Range	0.54 (0.363)	0.50 (0.970)	0.59 (0.199)	0.51 (0.932)	0.60 (0.425)

Rows are ordered by treatment-naive p-value; asterisks mark p<0.05. Kurtosis is the only feature significant in the treatment-naive stratum; the features significant treatment-blind (Entropy, Uniformity) or in a treated stratum (90th percentile, median, RMS, mean, 10th percentile — all in the liver-directed subgroup) are not significant in treatment-naive patients and are not carried forward.

**Figure 2:**
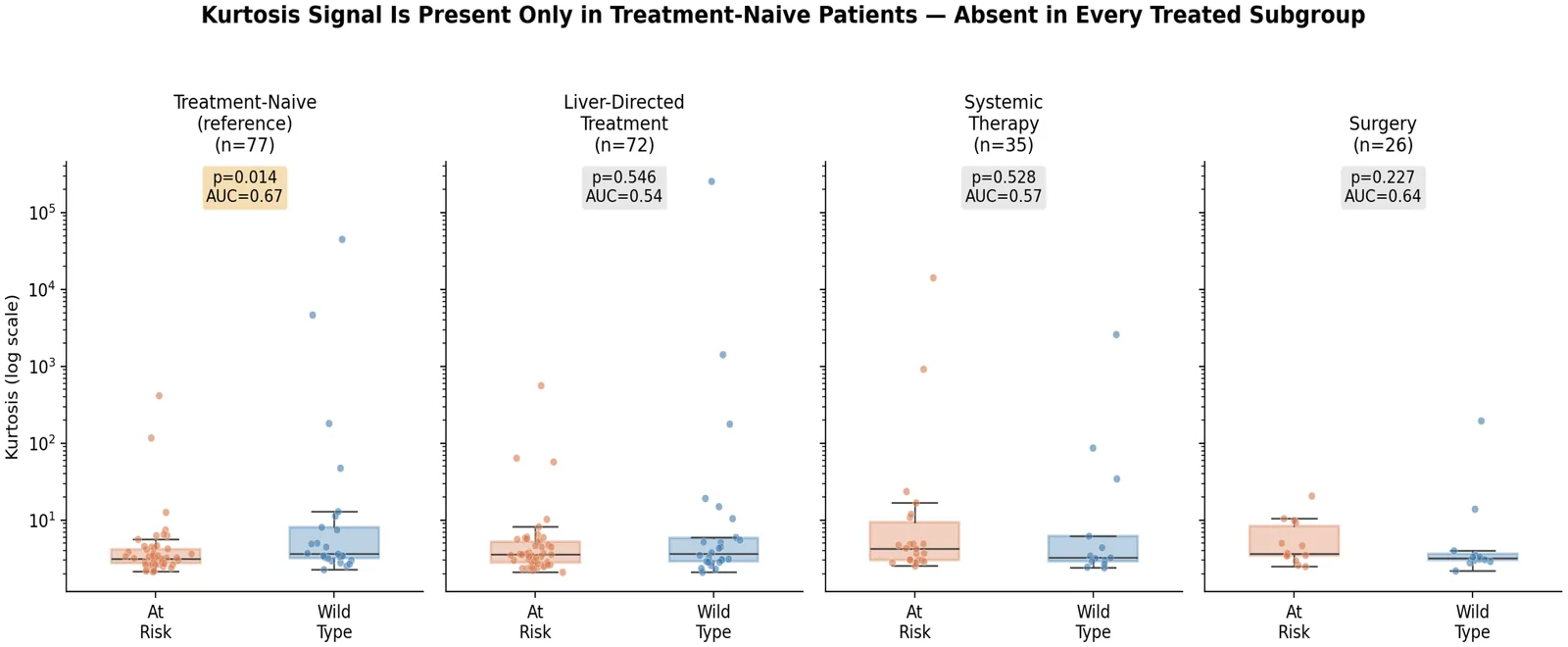
Kurtosis by genotype group: treatment-naive vs. each treatment modality analyzed independently. The genotype effect is present only pre-treatment and absent in every treated subgroup individually.

**Figure 3:**
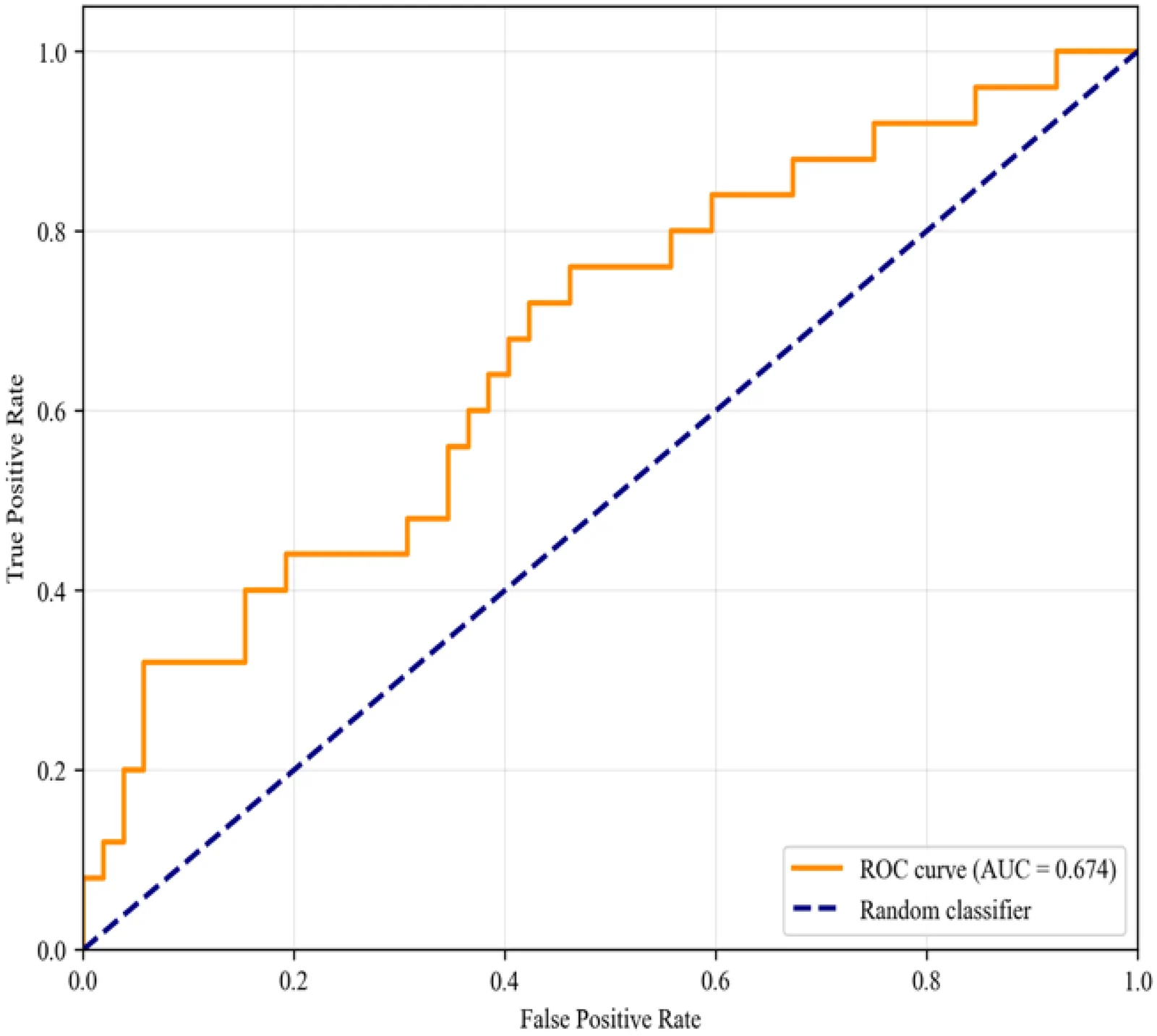
ROC analysis for kurtosis at-risk naive vs. wild-type naive (AUC = 0.674, 95% CI: 0.538–0.799).

### Kurtosis signal is robust to tumor-size and clinical confounding

Tumor volume correlated modestly with kurtosis across both genotype groups (Spearman ρ=0.45, p<0.0001; **Figure 4**), establishing a plausible mechanism by which tumor size alone could confound the genotype comparison: larger tumors can produce different intensity-distribution statistics for purely geometric reasons unrelated to genotype. To address this confounding, two complementary approaches were used: (1) regressing log-kurtosis on log-tumor-volume across the full cohort and testing the residuals for a genotype difference, and (2) a multivariable ANCOVA modeling the genotype effect jointly with volume and shape covariates.

**Figure 4:**
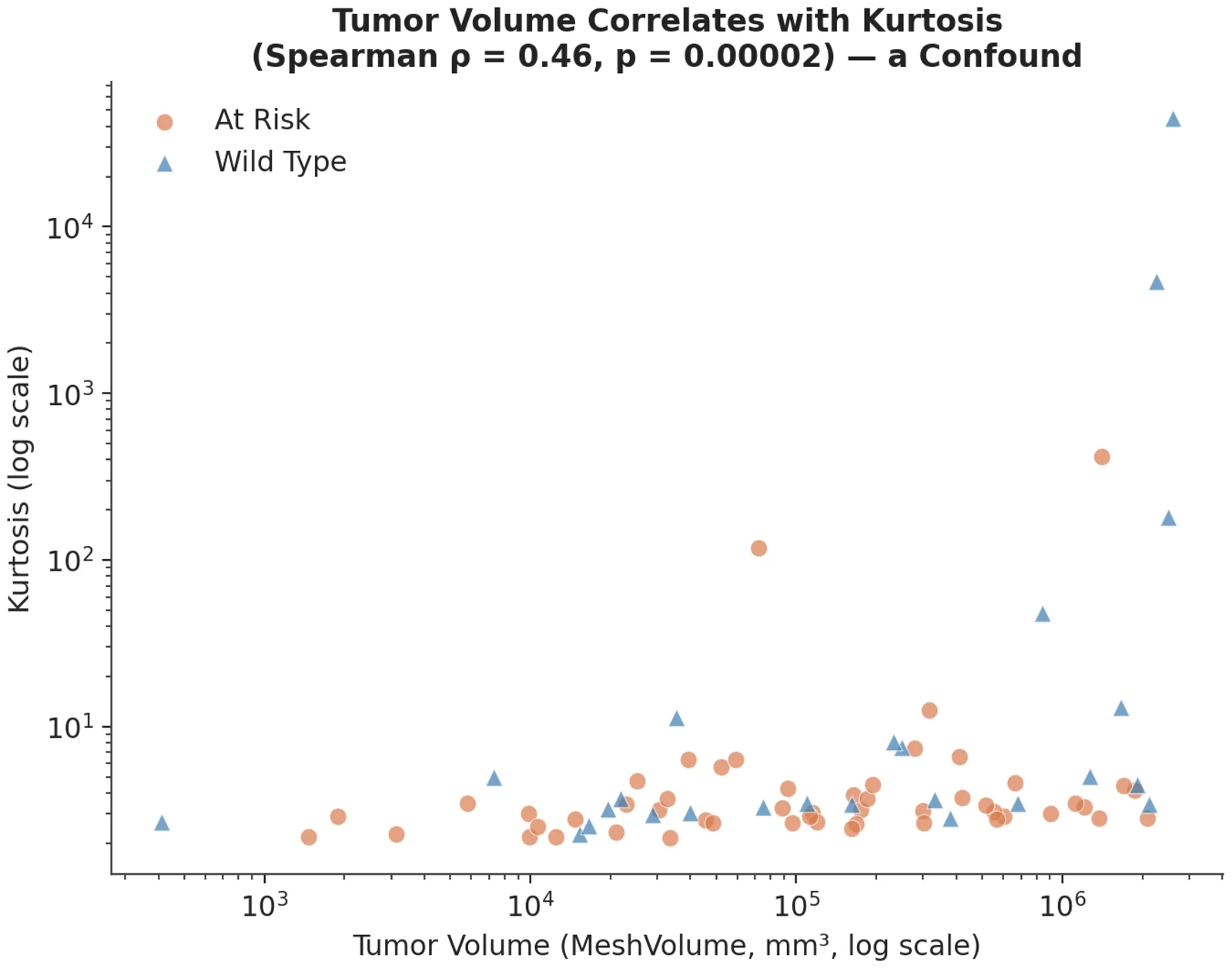
Kurtosis correlates with tumor volume across both genotype groups (Spearman ρ=0.45, p<0.0001).

In the first approach, log-kurtosis was regressed on log-tumor-volume across the full cohort, and the residuals (the component of kurtosis not explained by volume) were tested for a genotype difference using the Mann-Whitney U test. This comparison remained only borderline significant (p=0.057), indicating that volume accounts for some, but apparently not all, of the raw association. In the second approach, the multivariable ANCOVA modeling the genotype effect on kurtosis jointly with tumor volume, sphericity, and surface-to-volume ratio as covariates reached statistical significance (p=0.029; **Figure 5**). The ANCOVA constitutes a more appropriate and statistically more efficient test of the same hypothesis, as it estimates the genotype effect and the size/shape covariates simultaneously within a single model, rather than testing for a genotype difference in what remains after an independent, nonparametric adjustment step. Concordance in the direction of effect across both approaches, together with significance in the more appropriate model, supports the conclusion that tumor size and shape do not fully account for the observed genotype-kurtosis association. As an additional, independent check, logistic regression models predicting elevated kurtosis from genotype group, adjusted separately for log tumor volume (OR = 3.16, 95% CI 1.11–9.35, p=0.037) and for maximum 3D diameter (OR = 3.30, 95% CI 1.11–9.53, p=0.040), yielded concordant results (**Table 3**).

**Figure 5:**
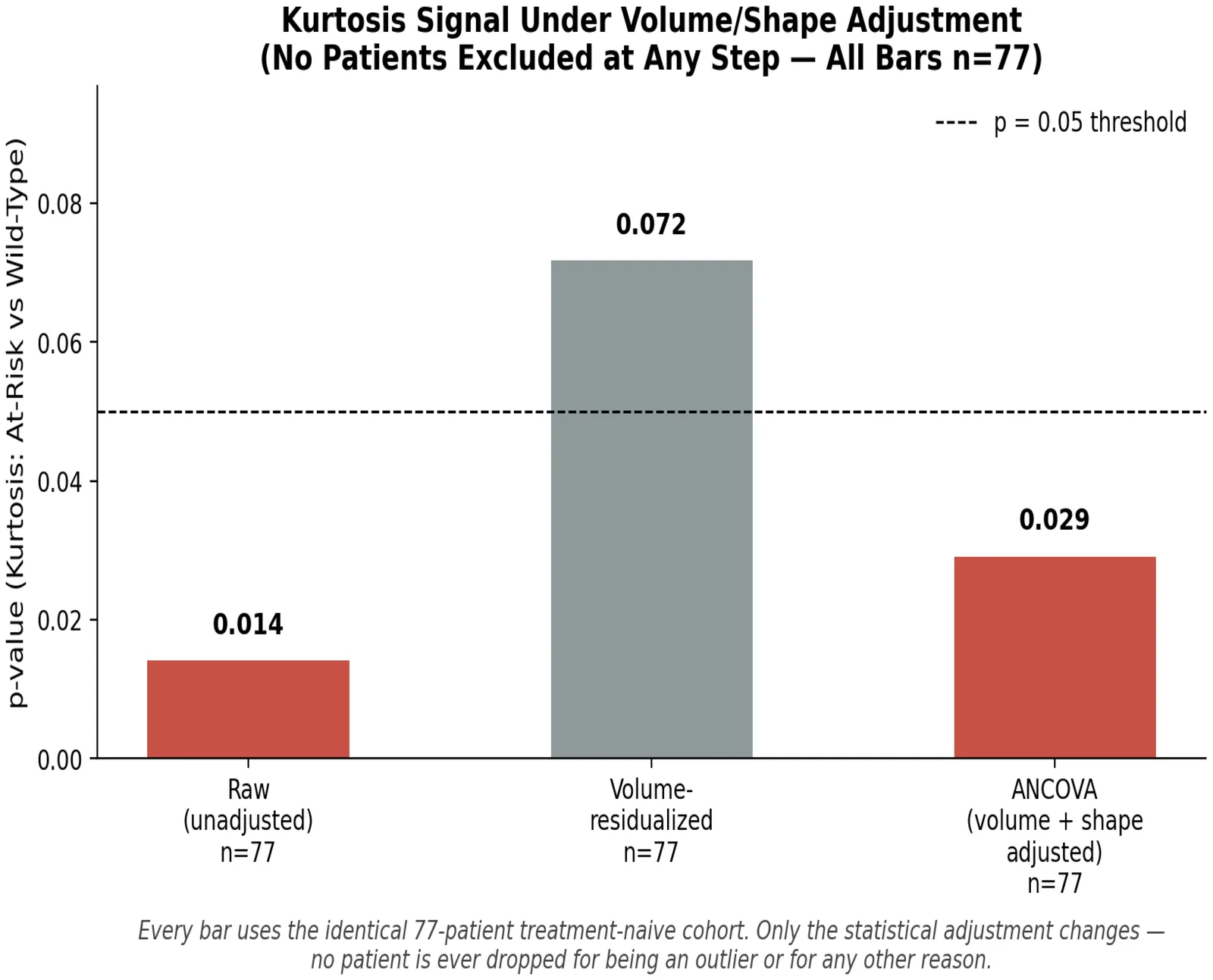
The kurtosis group-difference p-value under progressively stricter volume control, with no patients excluded at any step (all bars n=77): raw (p=0.014); after residualizing on log tumor volume (p=0.057); and after a full ANCOVA adjusting for tumor volume and shape simultaneously (p=0.029).

**Table 3:** Treatment-naive sensitivity analyses of elevated kurtosis (above empirical threshold) adjusted for tumor-size measures.

Model	Covariate(s)	OR (wild-type vs at-risk)	95% CI	P-value
Unadjusted threshold model	None	3.14	1.16 to 8.72	0.0260
Volume-adjusted threshold model	Log tumor mesh volume	3.16	1.11 to 9.35	0.0374
Diameter-adjusted threshold model	Maximum 3D diameter	3.30	1.11 to 9.53	0.0404

Logistic regression models (n=77) with elevated kurtosis as the outcome and genotype group (wild-type vs at-risk) as the predictor of interest.

In the clinical/demographic ANCOVA (n=68 complete cases [9 treatment-naive patients had some missing clinical data]), the genotype effect on kurtosis remained significant and, if anything, strengthened after adjustment (p=0.003; **Figure 6**). Of the nine clinical covariates tested, only sex reached significance on its own (p=0.02); because the at-risk group in this cohort was more heavily male than the wild-type group and male sex was independently associated with higher kurtosis, sex acted as a statistical suppressor rather than a confounder, partially masking rather than inflating the unadjusted genotype effect. No other clinical variable (diabetes, hepatitis C virus, hepatitis B virus core antibody status, smoking, alcohol use, or cirrhosis) was associated with kurtosis.

**Figure 6:**
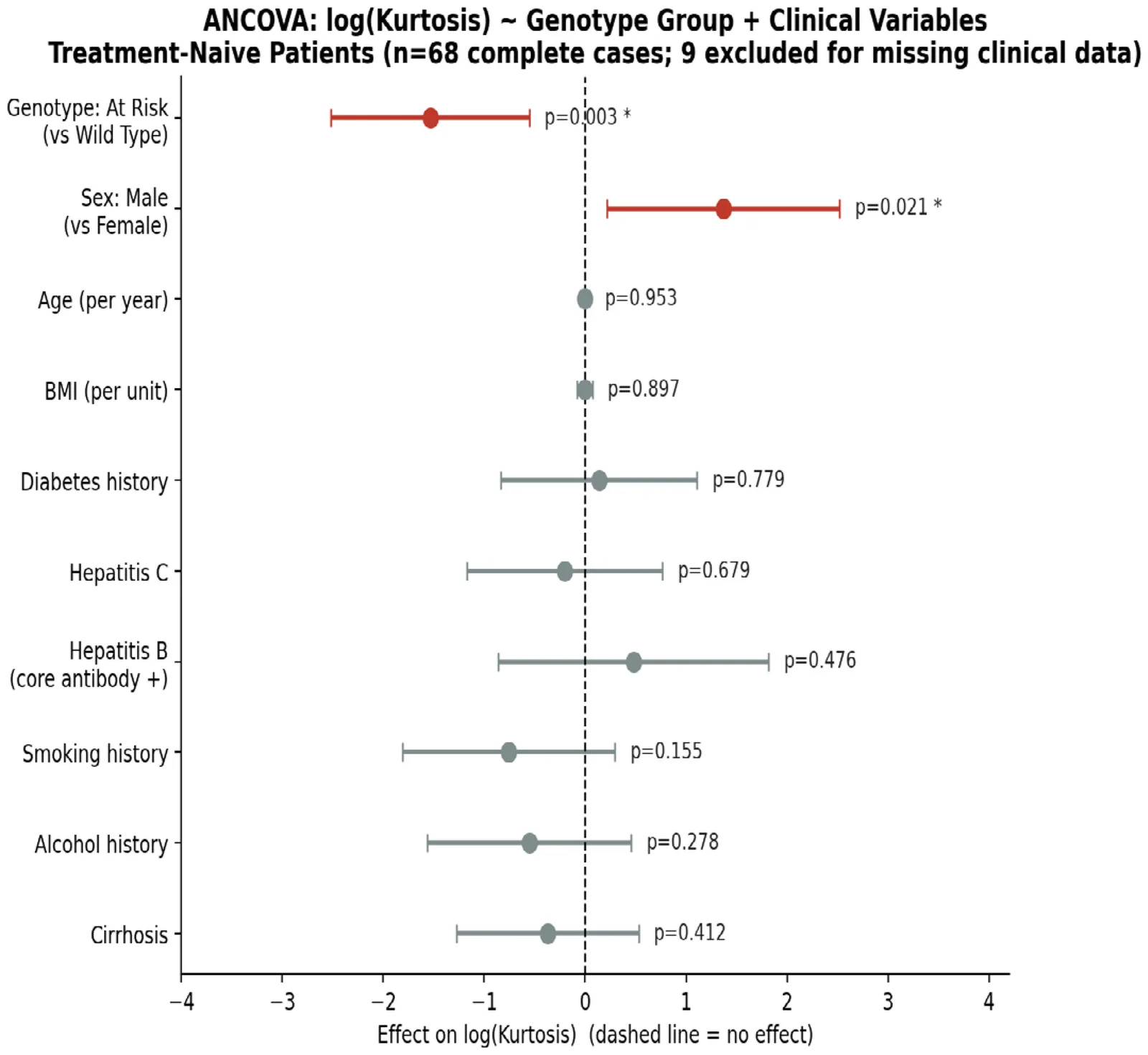
ANCOVA coefficients (effect on log-kurtosis) for genotype group and nine clinical/demographic covariates, fit simultaneously in one model.

### The empirical threshold is reproducible under cross-validation

Because the exploratory kurtosis cutpoint (**Table 4**) was derived and evaluated within the same cohort, we assessed its stability using 10-fold cross-validation. The median-based cutpoint, re-derived independently in each training fold, varied narrowly around the full-cohort value (mean=3.35, SD = 0.05), indicating that the threshold’s location is not an artifact of overfitting to a small number of patients (**Figure 7**). Out-of-fold odds-ratio estimates were directionally consistent with the full-cohort estimate in most folds but were more variable, reflecting the small size of each held-out fold (n≈8); we therefore report the threshold location as reproducible while treating the associated odds ratio as exploratory pending external validation.

**Table 4:** Exploratory contingency-table analysis of treatment-naive kurtosis using the empirical subgroup median threshold (>3.390), derived and evaluated within the same cohort.

Genetic group	Below threshold (≤3.390)	Above threshold (>3.390)	Total
At-Risk	31 (59.6%)	21 (40.4%)	52
Wild-Type	8 (30.8%)	17 (69.2%)	25
Total	39 (50.0%)	39 (50.0%)	77

**Figure 7:**
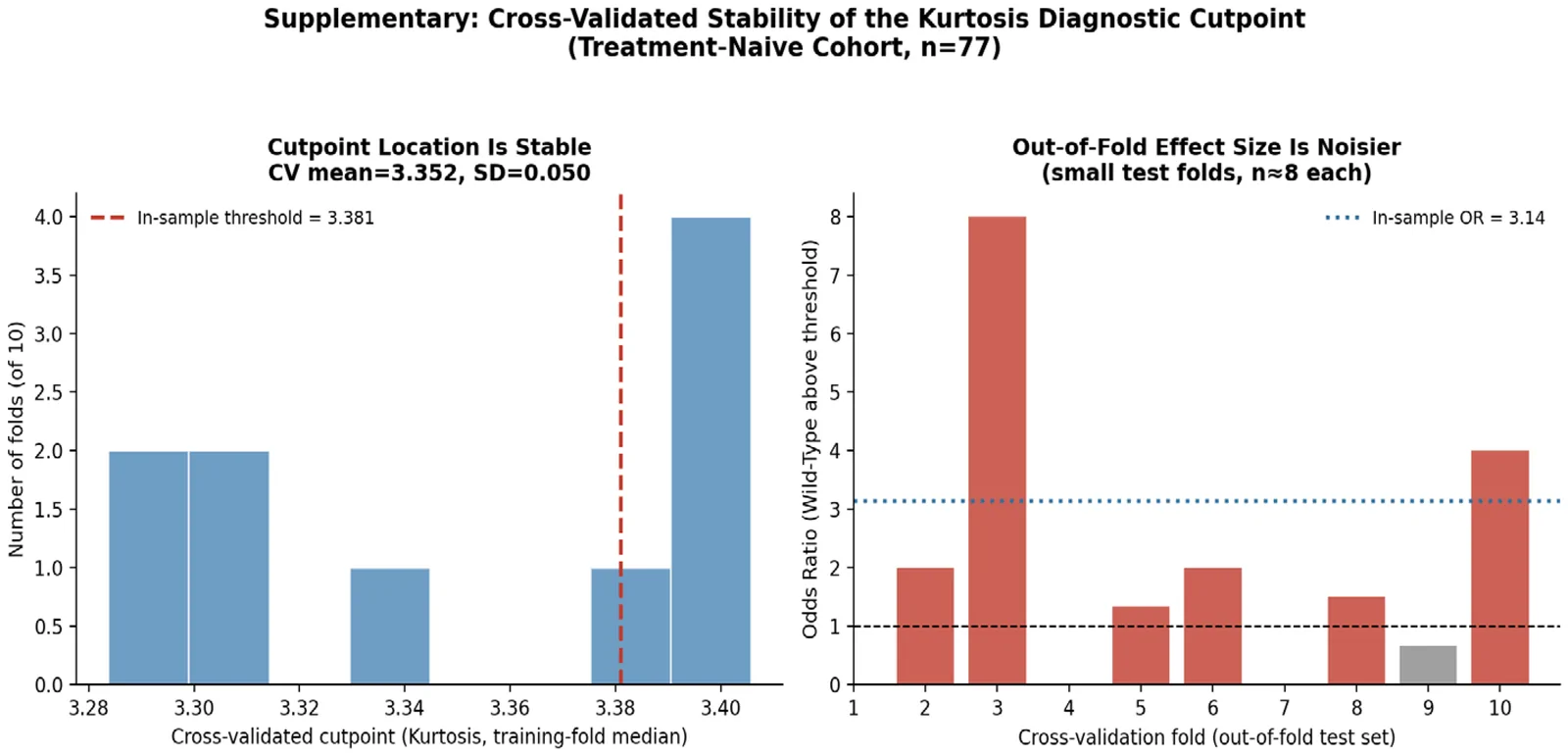
Left: the cutpoint location, re-derived independently in 10 training folds, is stable. Right: out-of-fold odds ratios are directionally consistent but noisier, reflecting small per-fold sample sizes.

### Kurtosis is not associated with overall survival

Kurtosis and genotype group were not significantly associated with overall survival in the treatment-naive cohort (Cox proportional-hazards regression per unit log-kurtosis: HR = 1.19, 95% CI 0.95–1.49, p=0.13; Kaplan-Meier comparison of high- versus low-kurtosis groups at the empirical cutpoint: p=0.18; **Figure 8**). We report this null result plainly: at this stage, kurtosis should be interpreted as a genotype-associated imaging phenotype rather than a validated prognostic biomarker.

**Figure 8:**
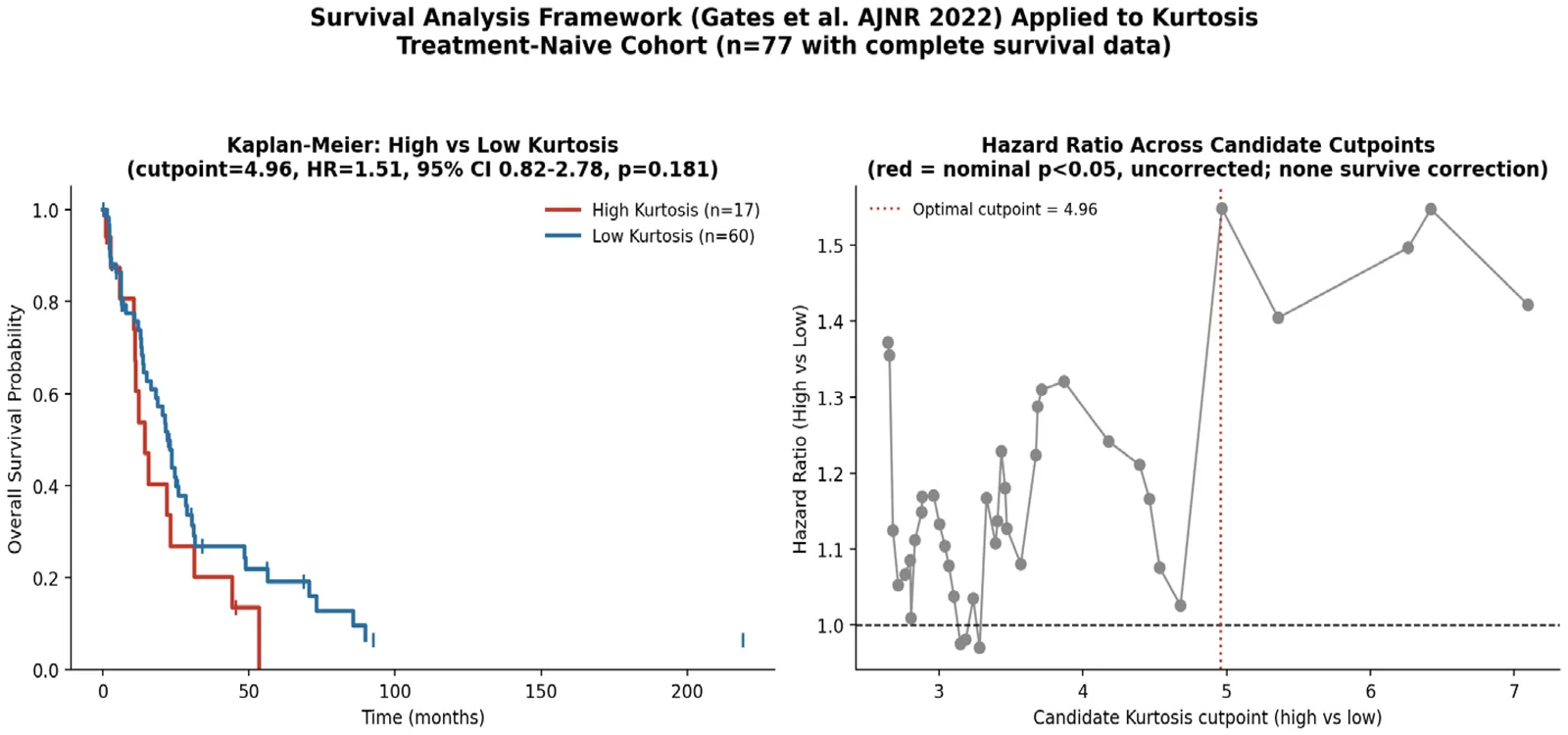
Left: Kaplan-Meier curves for high- vs. low-kurtosis patients at the empirical cutpoint (p=0.18). Right: hazard ratio across candidate cutpoints; none survive correction for multiple comparisons.

### Summary of key results

Treatment-naive patients demonstrate image-based genetic discrimination, with kurtosis emerging as the primary discriminator (raw p=0.014, OR = 3.14 at the empirical threshold). Wild-type patients develop tumors with more peaked intensity distributions, whereas at-risk patients exhibit broader intensity distributions. This association (1) is specific to untreated tumor tissue and absent within every individual treatment modality, (2) survives formal adjustment for tumor volume and shape (ANCOVA p=0.029) and strengthens after adjustment for nine clinical/demographic covariates (p=0.003), (3) is anchored to a threshold whose location is reproducible under 10-fold cross-validation, (4) is unchanged whether or not a single segmentation-outlier patient is included, and (5) is not associated with overall survival. Taken together, these results support kurtosis as a modest but genuinely confounder-tested imaging correlate of HCC genetic risk status in treatment-naive patients.

## Discussion

We analyzed 210 patients with HCC with the primary aim of identifying an association between genetic background and MRI imaging signatures, and the secondary aim of testing that association against the confounders most likely to explain it away. The central finding was a modest but reproducible association between genetic risk status and tumor kurtosis in treatment-naive patients (raw p=0.014; volume/shape-adjusted p=0.029; clinically adjusted p=0.003), which persisted across every confounding and robustness check we applied. This association provides novel insight into how genetic variants may influence tumor morphology on MRI.

Previous studies have suggested associations between specific genetic variants and imaging findings in HCC. For example, CTNNB1 mutations have been associated with specific enhancement patterns on contrast-enhanced imaging, while TP53 mutations correlate with certain morphological features (14, 15). However, these studies have typically focused on somatic mutations rather than germline variants, and systematic testing of confounding in genetic-imaging associations has been uncommon in this literature.

Kurtosis measures the degree of “peakedness” in intensity distributions within tumor regions. Because kurtosis is a first-order feature, it should not be interpreted as a direct measure of spatial homogeneity in the same way as texture-based radiomic features; rather, higher kurtosis indicates more tightly clustered voxel values around the mean, while lower kurtosis suggests broader intensity distributions with more values in the tails, indicating greater intensity dispersion and potentially more complex microenvironmental patterns (16). A previous study found higher kurtosis and lower skewness of non-cancerous liver parenchyma on MRI, suggesting that background parenchymal features in patients with HCC could predict future HCC development (17); this raises the possibility that the genotype-kurtosis association we observed reflects differences in vascularity, necrosis patterns, or cellular architecture between genetic groups, though tissue correlation studies would be needed to confirm this. Texture and intensity-based MRI features have also been shown to differentiate among hepatic lesion types and to distinguish HCC from benign lesions in prior work (18–20), supporting the broader premise that first-order and textural imaging features carry biologically meaningful information in liver tumors.

A recurring weakness in single-feature radiomic studies is that tumor size and clinical covariates are often acknowledged as potential confounders. Tumor volume was, as expected, correlated with kurtosis, and a volume-only adjustment (residualization) brought the association to the edge of significance. It is important to note that volume explains part, but not all, of the raw association. The more appropriate test, a full ANCOVA modeling volume and shape as covariates, showed the genotype effect persisting. Clinical and demographic covariates, tested the same way, did not weaken the association at all. Sex behaved as a suppressor rather than a confounder, which is itself informative about how the raw, unadjusted comparison likely understated rather than overstated the true effect.

Our focus on kurtosis as a univariate biomarker represents a deliberate methodological choice aligned with established principles of biomarker discovery and validation. Recent systematic reviews have exposed critical weaknesses in published radiomics studies; most lack external validation, demonstrate poor reproducibility across scanners and institutions, and show evidence of overfitting (21). Multi-feature models combining dozens of weakly correlated texture features through machine learning algorithms are particularly vulnerable to these problems. Single-feature biomarkers demonstrate superior generalizability across imaging protocols and scanner manufacturers; kurtosis is robust to many technical variations that destabilize complex texture features, can be calculated using widely available open-source software (PyRadiomics), and requires no proprietary algorithms. These practical advantages accelerate clinical translation and enable rapid external validation.

### Limitations

The single-center retrospective design may limit generalizability, and the moderate sample size (treatment-naive n=77) requires validation in larger, ideally multicenter cohorts. We directly tested the two confounders tumor volume/shape and clinical/demographic covariates and the genotype-kurtosis association persisted in each case; nonetheless, exploratory *post hoc* analysis found that the volume/shape-adjusted odds ratio, though robust to the segmentation-outlier sensitivity check, was more sensitive to individual high-leverage kurtosis values than the unadjusted or clinically adjusted comparisons. We therefore present that specific covariate-adjusted effect size as hypothesis-generating rather than a fully validated estimate. The empirical kurtosis threshold within this cohort is not an externally validated clinical cutoff, though its location was reproducible under cross-validation. Kurtosis was not associated with overall survival in this cohort and should currently be regarded as a genotype-associated imaging phenotype rather than a prognostic biomarker. Statistical testing used unadjusted p-values without formal multiple-comparison correction across the 18-feature screen, which increases the risk of a false positive; kurtosis’s status as the leading candidate should therefore be interpreted as hypothesis-generating pending independent replication. Finally, our genetic analysis considered only three established risk genes, which may miss important genetic interactions or additional protective and risk variants.

### Future research

Future research should prioritize external validation in independent, ideally multicenter, treatment-naive cohorts; mechanistic studies correlating tissue samples with imaging to clarify the biological basis of the genotype-kurtosis association; and investigation of additional HCC-associated genetic variants and protective alleles. Pathway analyses integrating transcriptomic data may help clarify the mechanism linking genetic background to imaging phenotypes.

## Conclusions

We identified tumor kurtosis as a modest but reproducible imaging correlate of established HCC genetic risk status in treatment-naive patients, with wild-type patients showing higher, more peaked voxel-intensity distributions than at-risk patients. Unlike many single-feature radiomic associations, this finding was directly tested against tumor-size confounding, clinical and demographic confounding, and cutpoint overfitting. Genetic risk variants therefore appear to be associated with measurable differences in HCC imaging phenotype in treatment-naive tumors, and this association remained significant after adjustment for measured confounders. These findings support kurtosis as a well-vetted, hypothesis-generating radiogenomic biomarker candidate warranting external, multicenter validation before clinical application.

## Data Availability

The data analyzed in this study is subject to the following licenses/restrictions: The Dataset is an internal MD Anderson dataset. Requests to access these datasets should be directed to DTFuentes@mdanderson.org.
